# A maternal hypoxia mouse model to study the effect of late gestational hypoxia on offspring lung outcomes

**DOI:** 10.3389/fphys.2025.1513703

**Published:** 2025-02-27

**Authors:** Thi-Tina N. Nguyen, Caitlin V. Lewis, Daniel Colon Hidalgo, Janelle N. Posey, Mariah Jordan, Timothy E. Porfilio, Maya R. Grayck, Clyde J. Wright, Cassidy Delaney, Eva S. Nozik

**Affiliations:** ^1^ Cardiovascular Pulmonary Research Laboratories, Department of Pediatrics and Department of Medicine, University of Colorado Anschutz Medical Campus, Aurora, CO, United States; ^2^ Department of Pediatrics, Division of Pediatric Critical Care, University of Colorado Anschutz Medical Campus, Aurora, CO, United States; ^3^ Department of Medicine, Division of Pulmonary and Critical Care, University of Colorado Anschutz Medical Campus, Aurora, CO, United States; ^4^ Department of Pediatrics, Division of Neonatology, University of Colorado Anschutz Medical Campus, Aurora, CO, United States

**Keywords:** prenatal hypoxia, late gestation, lung development, neonatal outcomes, pulmonary hypertension

## Abstract

Extremely preterm birth predisposes infants to bronchopulmonary dysplasia and associated pulmonary hypertension (PH). High altitude exposure during pregnancy has also been shown to worsen infant lung and pulmonary vascular outcomes. Animal models addressing the mechanisms for how maternal hypoxia impacts postnatal and adult lung and pulmonary vascular outcomes are lacking and development of a model to address this gap would enable new mechanistic studies. We hypothesize that late gestational hypoxia disrupts lung and pulmonary vascular development in the offspring, leading to abrupted lung development and PH in adulthood. Pregnant wild-type mice were exposed to hypobaric hypoxia at 505 mmHg, from day 16.5 of gestation until birth. Lung and pulmonary vascular outcomes were measured in juvenile and mature offspring. We found that late gestational hypoxia resulted in abrupted alveolar and pulmonary vascular development in juvenile offspring and that adult offspring showed persistent abrupted alveolar development as well as PH. This striking model will provide a new opportunity to determine mechanisms responsible for poor outcomes secondary to maternal hypoxia and assess important factors that increase susceptibility to adult diseases in former preterm infants.

## Introduction

Bronchopulmonary dysplasia (BPD), or chronic lung disease of infancy, is a major complication of extremely preterm birth, predisposing former preterm infants to impaired lung function and pulmonary hypertension (PH) into adulthood (Delaney et al., 2015; Heath-Freudenthal et al., 2022). Extremely preterm birth is defined as birth occurring before 28 weeks of gestation (Morgan et al., 2022). While the lungs of extremely preterm infants are susceptible to postnatal insults such as oxygen and mechanical ventilation, the maternal stress itself that led to preterm birth can also disrupt the developing lungs (Delaney et al., 2015). One recognized maternal stressor is hypoxia, most well-studied in pregnant women residing at high altitude. Exposure to high altitude throughout pregnancy can worsen neonatal outcomes for diverse reasons, including its effects on placental function, fetal growth, and lung development (Postigo et al., 2009; Julian, 2017; Moore, 2021; Gonzalez-Candia and Herrera, 2021; Fallahi et al., 2022; Heath-Freudenthal et al., 2024). Discerning the effects of maternal hypoxia during the vulnerable canalicular period on subsequent lung development could uncover novel insight into factors responsible for abrupted lung development in extremely preterm infants.

Several rodent models have examined the effects of antenatal, perinatal or postnatal hypoxia on placental or lung outcomes, however no model specifically examines how prenatal hypoxia impacts postnatal and adult outcomes. A series of studies have exposed pregnant mice to hypoxia (10%–13% FiO_2_) beginning at embryonic day 14.5 (E14.5) (Higgins et al., 2015; Cahill et al., 2017; Lane et al., 2020). At this timepoint, the fetal lungs are in the pseudoglandular phase of development, analogous to 7–17 weeks of gestation in human infants (Schittny, 2017; Chao et al., 2015; Hussain et al., 2017). In these studies, prenatal hypoxia disrupted placental morphology (Higgins et al., 2015), increased uterine artery blood flow (Lane et al., 2020), impaired fetal growth (Higgins et al., 2015; Lane et al., 2020), and reduced fetal lung pulmonary blood flow and lung volumes (Cahill et al., 2017). Other studies have exposed mice to hypoxia in the postnatal period and evaluated BPD and PH endpoints (Bierer et al., 2011; Mundo et al., 2021; Roberts et al., 2022). For example, exposure to postnatal hypoxia, from postnatal day 2–9, resulted in PH in the neonatal period (Bierer et al., 2011) and 2 weeks of postnatal hypoxia demonstrated BPD and PH phenotypes in 2-week-old mice (Roberts et al., 2022) while perinatal hypoxia, from E15 through postnatal day 4, led to PH in the adult offspring (Mundo et al., 2021). Collectively, these studies convincingly demonstrate that gestational hypoxia can impact fetal lung development and perinatal hypoxia can have long-standing effects into adulthood. These studies also present an opportunity to address a gap in knowledge as they do not test if late gestational hypoxia alone can disrupt lung and pulmonary vascular growth of the offspring into adulthood. This information is critical to advance the understanding of how maternal stress can impact both fetal and adult outcomes.

To address this gap, we sought to develop a model of maternal hypoxia to examine postnatal effects on lung and pulmonary vascular outcomes. We hypothesized that maternal hypoxia during late gestation disrupts lung and pulmonary vascular development in the offspring, leading to impaired lung structure and PH in adulthood. We exposed pregnant mice to hypobaric hypoxia (505 mmHg) from E16.5 until birth. We assessed somatic growth and alveolar and pulmonary vascular development in the immature and mature offspring, as well as PH endpoints in adult offspring.

## Materials and methods

### Late gestational hypoxia exposure

C57BL/6 mice were bred in-house in ambient Denver altitude. Female mice were examined for a mucus plug each morning to time the onset of pregnancy. On embryonic day (E)16.5, pregnant dams were randomly assigned to either normoxia (Denver altitude; 5,285 ft) or late gestational hypoxia. For hypoxia exposure, dams were placed in hypobaric hypoxic chambers simulating an approximate altitude of 11,500 ft above sea level (P_B_ ∼ 505 mmHg), equivalent to 13% fraction of inspired oxygen. P_B_ ∼ 505 mmHg was selected to mitigate fetal growth restriction and neonatal mortality (Mundo et al., 2021) that would impact lung development. Dams were checked each morning, and upon giving birth on E19.5, the dams and their pups were placed in room air. Pups from each litter were counted on the day of birth and postnatal day 4 (P4) for litter size and survival. Somatic growth was measured at P4 and P14. Offspring endpoints were assessed at P14 and 6 weeks. Nesting enrichments, water and food were provided *ad libitum*. Studies were repeated in three litters per group. Animal studies were approved by the University of Colorado Anschutz Medical Campus Institutional Animal Care and Use Committee.

### Tissue collection

Mice were euthanized at P14 or 6 weeks by carbon dioxide asphyxiation followed by cervical dislocation. Lungs were inflated with 1.5% LowMelt agarose (BioExpress; Cat No. E-3128-25). The volume of agarose was optimized for the two age groups at 400 µL for P14 whole lungs and 500 µL for 6-week-old left lungs. Following agarose inflation, crushed ice was placed on the lungs to polymerize the agarose for 3–4 min. Lungs were then removed and equilibrated in 4% paraformaldehyde (PFA) for fixation for 24 h, transferred to 10% neutral formalin buffer, embedded in paraffin, and sectioned longitudinally (5 µm). Sectioned lungs were then used for immunohistochemical staining.

### Immunohistochemistry

Immunohistochemistry was performed on paraffin-embedded lung tissue sections (5 µm). To identify proliferating cells, rabbit monoclonal Ki67 (1:200; Invitrogen, Carlsbad, CA; Cat: MA5-14520) was used with the Dako kit (Agilent, Santa Clara CA; Cat: K4065). Apoptosis was determined using the Abcam TUNEL Assay HRP-DAB kit (ab206386), according to the manufacturer’s instructions. For assessment of vessel density and muscularization, lung sections were co-stained for Von Willebrand Factor (vWF) (1:200; Thermofisher, Cat: PA5-80223) and α-smooth muscle actin (α-SMA) by the University of Colorado Anschutz CVP Histology Core Lab. Vessel density was determined as the total number of small vessels (<30 µm). Muscularization was defined as vessels (<30 µm) with >70% of the vessel wall positive for α-SMA. The ratio of muscularized to total vessel number was calculated. Analysis of proliferation, apoptosis, vessel density, and muscularization were manually counted on six to eight fields of view per lung within the distal region and measured by two blinded investigators.

### Lung morphometrics

H&E-stained lung sections were scanned with a whole slide scanner OlympusVS120 (Evident, Waltham, MA) using ×20 objective. Images were exported as .tiff using CellSense (Evident) software. Six to eight non-overlapping fields of view per lung section were assessed for alveolar development via radial alveolar count (RAC) using a manual protocol and mean linear intercept (MLI) using an image analysis software (Meta Series Software 7.8.13; Molecular Devices, LLC, Sunnyvale, CA), as previously described (Cooney and Thurlbeck, 1982; Nguyen et al., 2019; Dobrinskikh et al., 2021; Sherlock et al., 2022). RAC quantification was performed by two independent blinded investigators and an average of the two values was used.

### Hemodynamic measurements and right ventricular hypertrophy

Mice were anesthetized with isoflurane (2%–4%) and right ventricular systolic pressure obtained via direct right ventricle puncture in a closed chest with a 25-gauge needle as previously described (Tseng et al., 2020). To measure right ventricular hypertrophy, the right ventricle (RV) was dissected from the septum and the left ventricle (LV + S). Fulton’s Index, the ratio of RV weight to LV + S weight (RV/LV + S), was calculated.

### Statistical analysis

Data on litter size, percent survival, and offspring weight and length were presented for each pup as n = 1 pup and the data were collected from at least 3 litters. For immunohistochemistry and morphometric analysis, we analyzed n = 2–3 pups from each of three separate litters for each experiment. For RVSP and RVH data, we analyzed n = 2–7 mice from three separate litters. For additional analysis accounting for litter effects, an average of each litter per dam was used as n = 1 and presented in the Supplementary Material. Significance was determined by unpaired t-test using Prism v10 (Graphpad software, La Jolla, CA) and defined as p < 0.05. Data were expressed as the mean ± standard error.

## Results

### Late gestational hypoxia transiently impairs early somatic growth with recovery by day 14 of life

**FIGURE 1:**
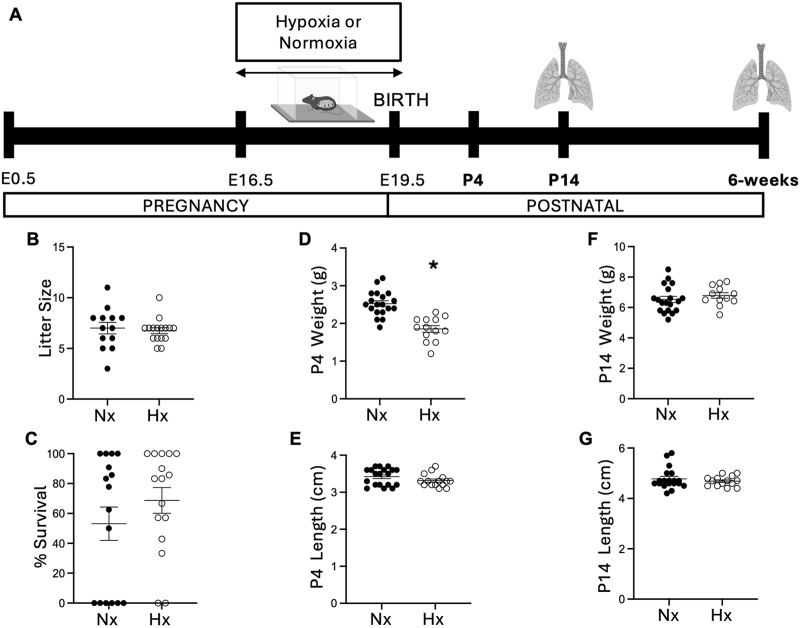
Exposure to late gestational hypoxia impairs early offspring somatic growth. Timeline of late gestational prenatal hypoxia exposure and endpoint analysis **(A)**. Timed pregnancy mice were placed into hypobaric hypoxic chambers (505 mmHg) or set at normoxia (room air; 633 mmHg) at E16.5 until E19.5. Pups were evaluated for litter size at birth **(B)** and percent survival **(C)** of each litter at P4; n = 12–16. Weight and length were measured in each pup at P4 **(D, E)** and at P14 **(F, G)**. Data analyzed by unpaired t-test, *P < 0.05. Each point represents an individual pup, n = 10–17 pups from three separate litters. Nx, normoxia; Hx, late gestational hypoxia.

Pregnant dams were placed in hypoxia from embryonic day 16.5 (E16.5) to 19.5 (E19.5), and offspring endpoints were measured at postnatal day 14 (P14) and at 6 weeks of age (Figure 1A). We observed no effect of hypoxia on litter size (Figure 1B), litter survival rate (Figure 1C), or maternal post-birth weights (Supplementary Figure S1). However, we found that offspring exposed to late gestational hypoxia weighed significantly less at day 4 compared to control mice (Figure 1D) but recovered by day 14 (Figure 1F). Hypoxia exposure did not affect body length at either time point (Figures 1E, G).

### Late gestational hypoxia leads to abrupted alveolar development and pulmonary vascular remodeling

**FIGURE 2:**
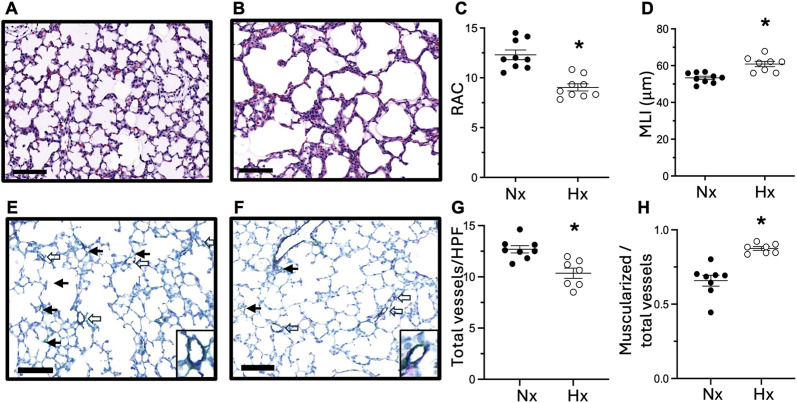
Exposure to late gestational hypoxia leads to abrupted alveolar and pulmonary vascular development in offspring at postnatal day 14. Representative H&E staining of lungs in offspring exposed to Nx **(A)** or late gestational Hx **(B)**. Quantification of radial alveolar count (RAC) **(C)** and mean linear intercept (MLI) **(D)**. Representative image of co-staining of vWF (green) for vessel density and α-sma (purple) for muscularized vessels in offspring exposed to Nx **(E)** or Hx **(F)** with enlarged image of muscularized vessels. Closed arrows are non-muscularized vessels and open arrows are muscularized vessels <30 μm, scale bar is 100 μm, ×20. Quantification of total number of small vessels by vWF **(G)**, and the ratio of muscularized small vessels to total number of small vessels **(H)**. n = 7–9 where each n represents individual pups selected randomly from three separate litters, with 2–3 pups tested per litter. Data analyzed by unpaired t-test, *P < 0.05.

To determine whether late gestational hypoxia impacts postnatal lung development, we evaluated lung structure at P14. In lungs stained with hematoxylin and eosin, we quantified alveolar development by radial alveolar count (RAC) and mean linear intercept (MLI) (Figures 2A–D). At P14, RAC was decreased and MLI was increased in offspring exposed to late gestational hypoxia, indicative of abrupted lung development. We evaluated pulmonary vascular remodeling by co-staining with Von Willebrand Factor and α-smooth muscle actin. At P14 pulmonary vessel density was decreased, and muscularization was increased in pups exposed to late gestational hypoxia (Figures 2E–H).

### Late gestational hypoxia blunts early cell proliferation and apoptosis in the lung

**FIGURE 3:**
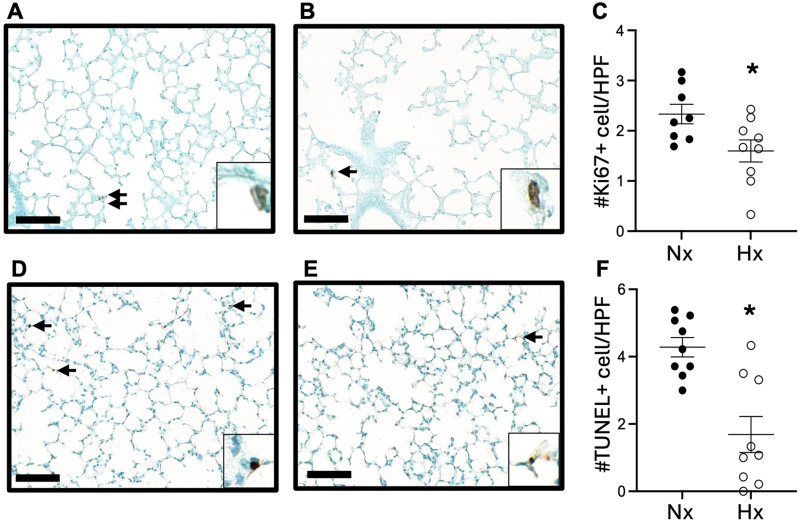
Exposure to late gestational hypoxia blunts early cell proliferation and apoptosis at P14. Representative image of Ki67 staining in offspring exposed to Nx **(A)** or Hx **(B)**. Quantification of cells positive for proliferation via Ki67 **(C)**. Representative image of apoptosis via TUNEL assay in offspring exposed to Nx **(D)** or Hx **(E)**. Quantification of cells positive for apoptosis via TUNEL assay **(F)**. Arrows indicate positive cells, scale bar is 100 μm, ×20; enlarged image of positive cell included in corner image. Six to eight fields of view were quantified per n. n = 8–9 where each n represents individual pups selected randomly from three separate litters, with 2–3 pups tested per litter. Data analyzed by unpaired t-test, *P < 0.05.

We next examined lung cell proliferation and apoptosis by immunohistochemical staining for Ki67 and TUNEL staining. At P14, we observed significantly fewer Ki67-positive cells (Figures 3A–C) and TUNEL-positive apoptotic cells (Figures 3D–F) in the lungs of offspring exposed to late gestational hypoxia. By 6 weeks of age, very few cells positive for Ki67 or TUNEL were detected in the lungs in either group (Supplementary Figure S4).

### Abrupted alveolar development persists into adulthood and is accompanied by pulmonary hypertension

**FIGURE 4:**
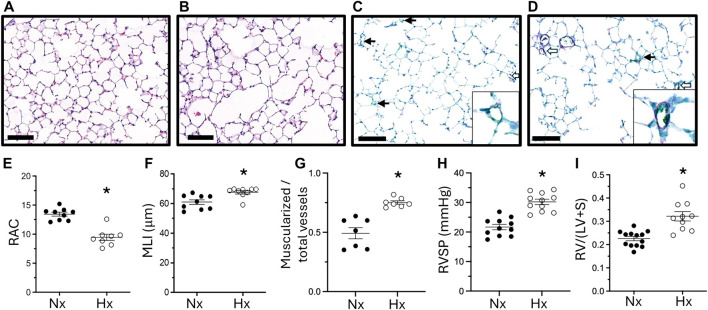
Abrupted alveolar development persists into adulthood and is accompanied by pulmonary hypertension. Representative H&E staining of 6-week-old lungs in offspring exposed to Nx **(A)** or Hx **(B)**. Quantification of RAC **(E)** and MLI **(F)**. Representative IHC of vWF (green) and α-sma (purple) co-staining in offspring exposed to Nx **(C)** or Hx **(D)** with enlarged image of a vessel in the lower right corner. Quantification of the ratio of muscularized/total number of small vessels **(G)**. Closed arrows are non-muscularized vessels, open arrows are muscularized arrows <30 μm, scale bar is 100 μm, ×20 magnification. Right ventricular systolic pressure (RVSP) **(H)** assessed via direct right heart puncture and Right Ventricle Hypertrophy **(I)** measured via right ventricle divided by left ventricle plus septum weight. n = 7–13 where each n represents individual pups selected randomly from three separate litters. Data analyzed by unpaired t-test, *P < 0.05.

Finally, we examined whether lung and pulmonary vascular abnormalities following late gestational hypoxia persisted into adulthood. Six-week-old mice exposed to late gestational hypoxia showed persistent impairment in alveolar structure, quantified by a significantly lower RAC and increased MLI (Figures 4A, B, E, F). Late gestational hypoxia also led to pulmonary vascular remodeling and PH at 6 weeks of age, demonstrated by increased muscularization of small vessels, increased RVSP and RVH (Figures 4C, D, G–I).

## Discussion

Current published models of maternal hypoxia have not examined the effects of maternal hypoxia on postnatal lung and pulmonary vascular outcomes. We thus developed a model of late gestational hypoxia and examined lung and pulmonary vascular development in the offspring at 14 days and 6 weeks. We demonstrated that late gestational hypoxia beginning at E16.5 in mice leads to abrupted alveolar and pulmonary vascular development, blunted early cell proliferation and apoptosis in immature lungs, and persistent impaired lung structure as well as pulmonary hypertension in adult mice.

In designing this new model of late gestational hypoxia, there were two key parameters to consider: the timing of the prenatal exposure and selection of the injurious stimulus. We initiated the exposure at gestational age E16.5, when the fetal lungs are in the canalicular stage of development, as the initial time for hypoxic exposure. The canalicular stage of lung development correlates with human fetal lung development of 17–26 weeks of gestation (Chao et al., 2015; Hussain et al., 2017). This is clinically significant because lung development and lung function determine the limits of viability following preterm birth, with the highest risk of extremely preterm infants born with immature lungs in the canalicular stage (Chao et al., 2015; Dumpa and Bhandari, 2021). Our protocol therefore provides an opportunity to model maternal stress during this vulnerable period and evaluate its effect on postnatal lung outcomes. There are several published studies that have also exposed pregnant mice to hypoxia. Lane et al. tested a more severe degree of hypoxia for a longer period in mice (385 mmHg to simulate 10% O2 from E14.5 to E18.5) and examined the effects of maternal hypoxia on fetal and uteroplacental outcomes, reporting impaired fetal growth as well as increased uterine artery blood flow (Lane et al., 2020). In addition, Mundo et al. exposed mice from late gestation (E15) to postnatal day 4 and evaluated adult lung outcomes, observing PH (Mundo et al., 2021). Our model advances the field as it limits the hypoxic exposure to late gestation but evaluates outcomes in the offspring into adulthood. Exposure to hypoxia is well-known to impact the lungs of mature and immature animals and humans (Stenmark et al., 2006; Pugliese et al., 2015; Shimoda, 2020; Gassmann et al., 2020). Postnatal hypoxia leads to PH, as well as abrupted lung development (Inscore et al., 1991; Bierer et al., 2011; Roberts et al., 2022) while chronic hypoxia in adult models leads to PH and pulmonary vascular remodeling (Villegas et al., 2013; Tseng et al., 2020; Colon Hidalgo et al., 2024). Though hypoxia can have a direct effect on lung development and induce PH, since it can also impair uterine artery blood flow and placental weights (Lane et al., 2020), it is unclear if the effects of hypoxia on offspring are due to a direct effect of hypoxic conditions on the developing lungs or due to impaired placental function. We do not suspect there was impaired maternal nutrition, as the weights of the mothers after birth were similar in both groups. Overall, we developed this model to focus on hypoxic stress in a vulnerable period of fetal lung development and evaluate juvenile and adult outcomes.

Our key observations were that late gestational hypoxia impaired alveolar and pulmonary vascular growth in juvenile mice, and lung and pulmonary vascular abnormalities persisted into adulthood as reduced alveolar septation and PH. The concurrent impact on both alveolar and pulmonary vascular development is consistent with the concept that alveolarization and pulmonary vasculature development are tightly coordinated in the developing lung (Abman, 2001). Abrupted lung development is further supported by the reduced cell proliferation and apoptosis observed in the developing lungs of 14-day-old offspring exposed to late gestational hypoxia. We did not see differences in cell turnover in the 6-week-old mice and attributed this finding to the fact that alveolarization was complete at this stage and thus there was low proliferation and apoptosis. Our lab and others have previously reported a transient increase in cell proliferation in the pulmonary vascular wall with 3 days of hypoxia in adult mice (Dempsey et al., 2009; Nozik-Grayck et al., 2008). In contrast, our current finding of blunted cell proliferation and decreased vessel density with late gestational hypoxia is consistent with abrupted lung development. Our observations demonstrate distinct mechanisms in the developing lung, and we propose that the increased muscularization at 14 days and 6 weeks may be a response to elevated pulmonary artery blood pressure associated with the abrupted vascular development. This remains to be investigated in future studies. Our data is overall consistent with *Barker’s Hypothesis* of fetal origins of disease, which states that chronic disease during adulthood originates from events during the fetal period (Barker, 2004). Of note, both preterm birth and prenatal hypoxia in humans are associated with adult lung and pulmonary vascular disease. Our findings align with existing studies demonstrating that young adults born prematurely develop pulmonary hypertension due to impaired right ventricular function and increased susceptibility to cardiovascular disease (Goss et al., 2018; Lewandowski et al., 2020; Barton et al., 2021). Multiple studies have shown that individuals born from perinatal hypoxia (i.e.,: high-altitude living in Bolivia) develop greater risk of pulmonary vascular dysfunction (Julian et al., 2015; Heath-Freudenthal et al., 2022; Heath-Freudenthal et al., 2024). We also found it very interesting that the fetal lungs responded substantially differently to hypoxia than the published response of the adult mouse lung. A brief period of hypoxia during late gestation led to sustained changes in the lung and pulmonary circulation while adult mice with hypoxia exposure do not demonstrate PH until 14–21 days (Villegas et al., 2013; Pugliese et al., 2017; Tseng et al., 2020) and the response reverses over time in adult mice once they are returned to normoxia (Pugliese et al., 2017). This observation could indicate that there are epigenetic changes occurring *in utero* that lead to long-standing consequences into adulthood. Epigenetic regulation is implicated in other models of maternal stress and fetal outcomes. For example, increased pulmonary DNA methylation is induced in the offspring of stressed mothers and these epigenetic changes can be passed on to the subsequent generation (Rexhaj et al., 2011). Maternal stress related to diet restriction was also shown to alter endothelial cell gene expression and function in the offspring pulmonary vasculature that is associated with pulmonary vascular remodeling (Zelko et al., 2019). Future studies will therefore investigate whether epigenetic modifications with late gestational hypoxia contribute to abrupted pulmonary vascular and alveolar development.

In conclusion, we developed a new model of late gestational hypoxia that adds to growing literature on the lasting impacts of maternal stress on postnatal outcomes. This late gestational hypoxia exposure model affected early somatic growth and led to abrupted lung development that persisted into adulthood. These findings provide new opportunities to learn about hypoxia exposure during the canalicular stage of fetal lung development and potential mechanisms of abrupted alveolar development and induced PH that are affected into adulthood, along with exploring therapeutic targets to attenuate these lung outcomes.

## Data Availability

The original contributions presented in the study are included in the article/Supplementary Material, further inquiries can be directed to the corresponding author.
