# Supplementary material for: A maternal hypoxia mouse model to study the effect of late gestational hypoxia on offspring lung outcomes
Source: Front Physiol. 2025 Feb 27;16:1513703. doi: 10.3389/fphys.2025.1513703 (PMC11904635; doi:10.3389/fphys.2025.1513703)
Supplement: Supplementary file 1 [file DataSheet1.pdf]

A

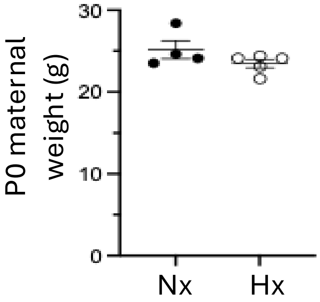

SFig 1A. Maternal weight post-birth (P0) with and without exposure to late gestational hypoxia. n = 4 – 5 dams per exposure group. Data analyzed by unpaired t-test, *, $P<0.05$.

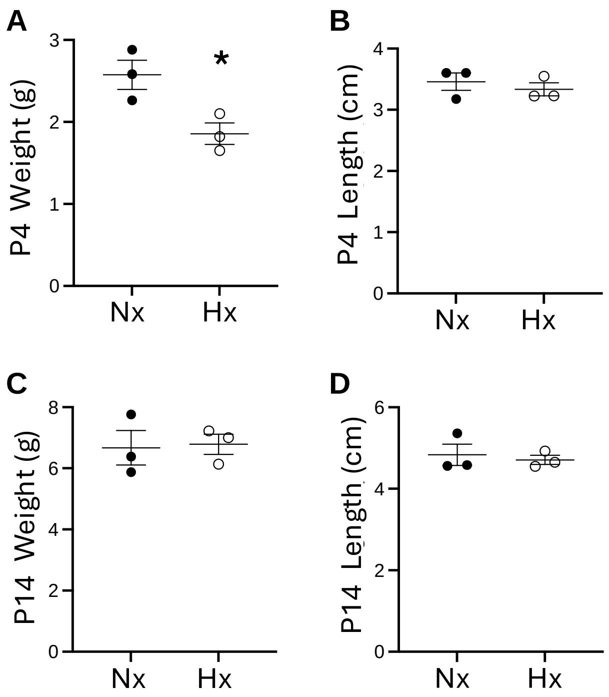

SFig 2. Somatic growth of offspring with exposure to normoxia and late gestational hypoxia. n = 1 is identified as the average weight and length of litter at P4 **(A,B)** and P14 **(C,D)** in reference to Figure 1 **(D,F,E,G)** from three individual litters per exposure. Data analyzed by unpaired t-test, *, $P<0.05$.

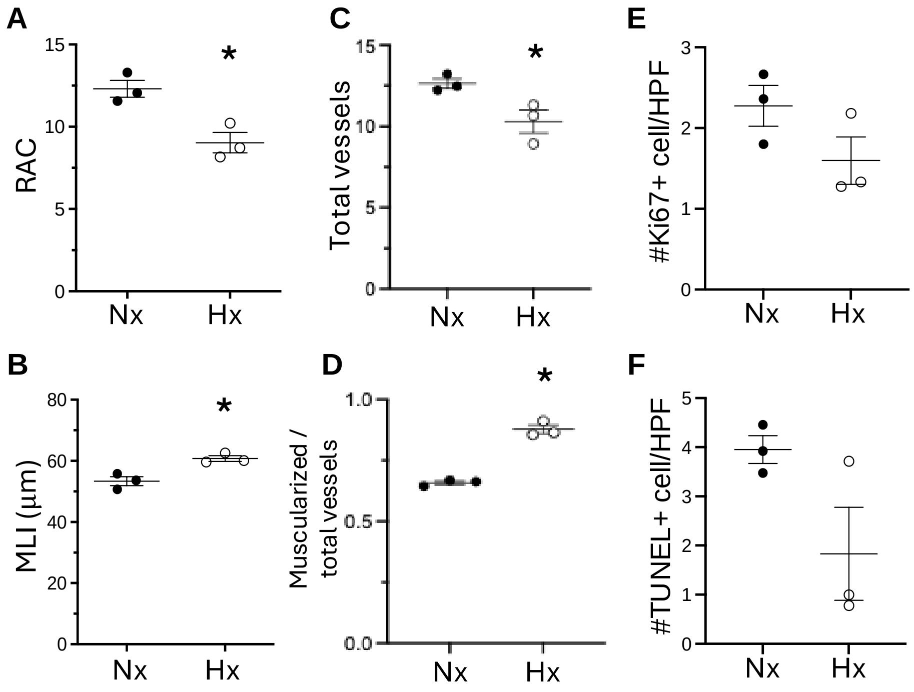

SFig 3. Alveolar and pulmonary vascular development and cell growth of offspring with exposure to normoxia and late gestational hypoxia at P14. n = 1 is identified as the average litter of RAC **(A)**, MLI **(B)**, vessel density **(C)**, ratio of muscularized to total vessels **(D)**, and cells positive for Ki67 **(E)** and TUNEL staining **(F)** in reference to Figure 2 and 3 from three individual litters per exposure. Data analyzed by unpaired t-test, *, $P<0.05$.

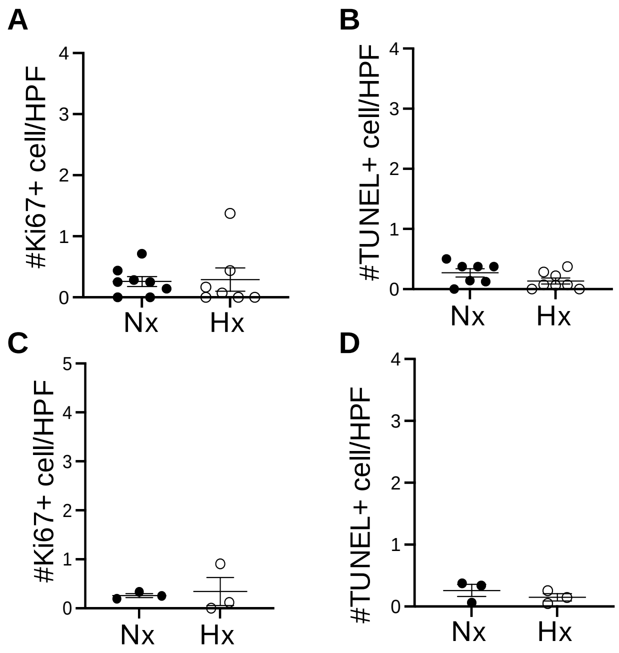

SFig 4. Lung cell growth with exposure to normoxia and late gestational hypoxia during adulthood. Proliferation via Ki67+ staining **(A)** and apoptosis via TUNEL+ assay **(B)** from n = 7 – 8 pups where each n represents individual pups selected randomly from three separate litters, with 2-3 pups tested per litter. Average of litter from cells positive for proliferation in Fig S4A **(C)**, and apoptosis in in Fig S4B **(D)**; n = 1 is identified as the average of positive cells per litter from three individual litters per exposure. Data analyzed by unpaired t-test, *, P<0.05.

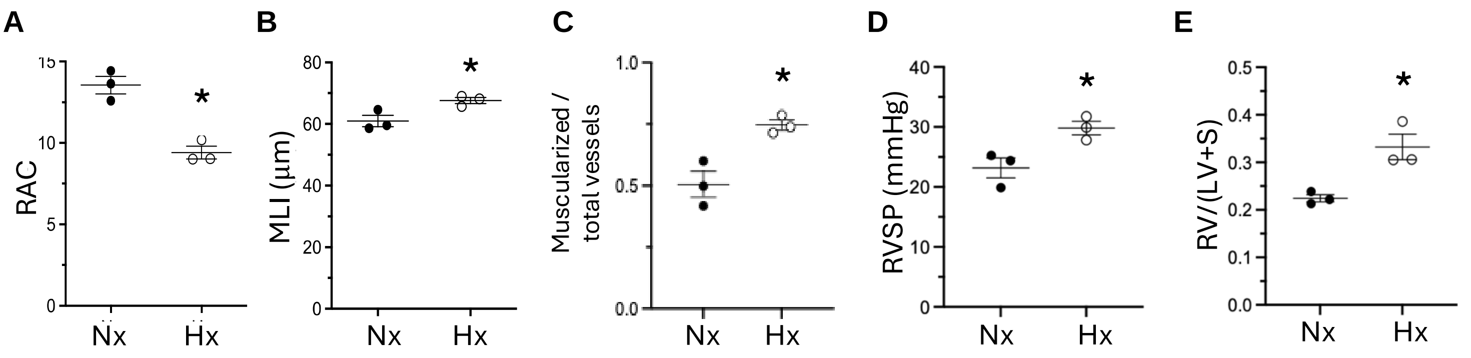

SFig 5. Impairment of alveolar and pulmonary vascular development persisted during adulthood, along with induced pulmonary hypertension with exposure to late gestational hypoxia. n = 1 is identified as the average litter of RAC **(A)**, MLI **(B)**, ratio of small muscularized vessels to total vessels **(C)**, RVSP **(D)**, and RVH **(E)** in reference to Figure 4 from three individual litters per exposure. Data analyzed by unpaired t-test, *, $P<0.05$.
